# Detection by Flow Cytometry of Anti-DNA Autoantibodies and Circulating DNA Immune Complexes in Lupus Erythematosus

**DOI:** 10.1155/2019/6047085

**Published:** 2019-12-05

**Authors:** Nisen Abuaf, Chantal Desgruelles, Mohamed Moumaris, Faïza Boussa-Khettab, Hidayeth Rostane, Emilie Bellec, Camille Frances

**Affiliations:** ^1^Laboratoire d'Hématologie et d'Immunologie, Hôpital Tenon, Groupe Hospitalier Universitaire Paris Est, AP-HP et Département d'Immunologie, Université Pierre et Marie Curie, 4 Rue de la Chine, 75020 Paris, France; ^2^Service de Dermatologie et d'Allergie, Hôpital Tenon, Groupe Hospitalier Universitaire Paris Est, AP-HP et Université Pierre et Marie Curie, 4 Rue de la Chine, 75020 Paris, France

## Abstract

A new method for the detection by flow cytometry of anti-double-stranded DNA antibodies and of circulating immune complexes (IC) containing endogenous DNA (IC-eDNA) is described. From each serum sample, two samples were taken, one was used to detect IC-eDNA. The other to detect anti-DNA antibodies was incubated with calf thymus DNA. ICs were isolated by polyethylene glycol precipitation or by cryoprecipitation, after which immunoglobulins were labeled with FITC-conjugated anti-human globulin. Serum samples from 63 systemic lupus erythematosus (SLE) patients, 32 incomplete lupus, and 87 control patients were tested. Detection of anti-dsDNA antibodies by flow cytometry had a diagnostic sensitivity and specificity almost comparable to routine tests, the fluorescent enzyme immunoassay EliA™-dsDNA test, and the ultrasensitive Crithidia luciliae indirect immunofluorescence test. In 21 (33%) out of 63 SLE serum samples, IC-eDNA was detected. In these samples, free anti-dsDNA antibodies were hardly detectable or undetectable by flow cytometry or by routine tests. When anti-DNA antibodies are neutralized by endogenous DNA and can no longer be detected by routine tests, the serologic diagnosis and the follow-up of relapses in patients with SLE is compromised. To overcome this obstacle, we propose an accessible solution: the detection of circulating IC-eDNA by flow cytometry.

## 1. Introduction

Antibodies to double-stranded DNA (anti-dsDNA) are the serological hallmark of systemic lupus erythematosus (SLE). The increase in the titer of anti-dsDNA antibodies concomitantly with decreased levels of complement proteins C1q, C3, and C4 is frequently associated with acute exacerbations of the disease because anti-dsDNA antibodies can mediate tissue inflammation by the formation of immune complexes (ICs) with endogenous DNA (IC-eDNA). Considerable evidence supporting the role of IC-eDNA in the pathogenesis of lupus nephritis has accumulated [1–9]. Therefore, according to the Systemic Lupus International Collaborating Clinics classification criteria (SLICC), a biopsy confirmed nephritis compatible with SLE associated with anti-dsDNA antibodies is sufficient for SLE diagnosis [2]. Numerous studies demonstrated the ability of ICs to effectively activate Toll-like receptors (TLR) and induce interferon (IFN) production [3, 4]. Enhanced IFN-I level in patients with SLE deregulates inflammasomes [5]. In the NOD-like receptor family, pyrin domain containing 3 and 1 (NLRP3 and NLRP1) inflammasomes are molecular platforms that detect the damage or danger signals of cells. ICs can activate the NLRP3 inflammasome [6]. Work in several murine models suggests an important role for the NLRP3 inflammasome in mediating lupus nephritis [7].

In serum samples of patients with SLE having active renal disease, a significant increase in the titer of anti-dsDNA antibodies was observed after DNase digestion. This indicates that in serum samples of these patients, eDNA had bound in vivo to the anti-dsDNA antibodies to generate circulating IC-eDNA [8]. The amount of DNA in the circulating IC-eDNA is highly correlated with disease activity [9].

Tan et al. in 1966 were the first to show the presence of eDNA in the human circulatory system of SLE patients [10]. DNA was found in the sera of 11 of 95 (12%) patients with SLE. The technique that had been used, the double immunodiffusion in agarose, was not very sensitive. Before this publication, Mandel and Métais had already published a paper in 1948 demonstrating DNA and RNA in the blood of healthy individuals and patients, but the clinical relevance was ignored [11]. The characteristics of circulating eDNA in SLE patients have been extensively studied. The eDNA may be present as oligonucleosomes, nucleosomes, chromatin, and immune complexes or included in particulate structures (exosomes, microparticles, apoptotic bodies) [9, 12–17]. Circulating IC-eDNA was isolated from serum samples of patients with clinically active SLE by ultracentrifugation in a sucrose gradient [12], by polyethylene glycol (PEG) precipitation [13], or alternatively DNA extracted from plasma with phenol-chloroform and proteinase K treatment [14].

Flow cytometry (FCM) was used to detect anti-dsDNA antibodies using microparticle-bound eDNA as the antigen [18, 19]. Subsequently, it was shown that FCM can also detect large ICs independently of microparticle-bound antigen or antibody [20, 21]. Using this data, we developed a sensitive FCM-based assay that evaluates the amount of circulating anti-dsDNA antibodies and of the circulating IC-eDNA. We compared this new dual screening test to the usual anti-dsDNA antibody detection tests and attempted to deduce the additional contribution to the diagnosis of SLE patients.

## 2. Patients and Methods

### 2.1. Serum Samples and Patients

63 patients satisfied the revised criteria for the classification of SLE of the American College of Rheumatology (ACR) and SLICC criteria [2, 22, 23]. Twenty-nine patients had incomplete SLE and satisfied <4 ACR criteria: 9 lupus nephritis, 14 chronic cutaneous lupus, and 6 subacute cutaneous lupus. Three patients had drug-induced lupus, 2 had received infliximab, and another anticancer chemotherapy. All patients included in this study were treated. For all patients, at least two serum samples at different dates were available. Thus, a longitudinal and cross-sectional analysis of anti-DNA antibodies in the samples could be performed.

87 patients with miscellaneous diseases except connective tissue diseases were included in the control group. Patients with more than one ACR or SLICC criteria were excluded. The patients were included in the study between 2014-2017. In the study approval, the study protocol was in accordance with the local ethical committee guidelines. Consenting patients have undergone blood samples when the detection of the anti-DNA antibodies was needed for diagnosis or for follow-up. Serum samples were conserved at 4°C.

### 2.2. Chemicals and Reagents

Deoxyribonucleic acid sodium salt from calf thymus type I fibers (ref D1501) were purchased from Sigma-Aldrich, 3050 Spruce Street, Saint Louis, MO 163103, USA. The product was dissolved in 2 mg/ml in water and maintained overnight at 4°C to completely solubilize the DNA. After which, aliquots are conserved at -40°C.

Bovine deoxyribonuclease I (DNase I) from bovine pancreas (ref D4263) from Sigma-Aldrich was tested between 0.2 and 0.02 mg/ml with 5 mM Ca^2+^ and 5 mM Mg^2+^.

Tween 80 and Triton X-100 and polyethylene glycol MW 6000 (PEG) were purchased from Prolabo, Rhône Poulenc, 12 Rue Pelée, 75011 Paris, France. Detergents were used 1/1000 at dilution. PEG was used at final concentration of 3.5%. DNAse I or detergent treatment of samples was done during 30 min before adding PEG.

Ethidium bromide (EB) 10 mg/ml, molecular biology grade (ref 11ETBC1001), was purchased from MP Biomedicals SARL, Parc d'Innovation BP50067, Rue Geiler de Kaysersberg, Illkirch Cedex 67402, France. It was used 1/1000 at final dilution. EB is used in one-step staining protocol for chromosomes flow cytometric DNA analysis [24, 25].

EB solutions were handled with extreme caution; the waste was inactivated with bleach (sodium hypochlorite) and collected by a factory specialized in the processing of wastewater with chemical hazard, as was recommended [26].

FITC-conjugated antiserum to human immunoglobulins (IgG, IgA, IgM) produced by Kallestad, with 1% bovine serum albumin and 0.1% sodium azide. Liquid conjugate at the working dilution (ref 30480 for100 ml or 30446 for 2.5 ml) was purchased from Bio-Rad Laboratories, 3 Boulevard Raymond Poincaré, 92430 Marnes-la-Coquette. In order not to introduce any difference between the methods due to the labeling of the different antibody isotypes, the same antiglobulin was used in FCM, in the Crithidia luciliae indirect immunofluorescence test and for the detection of the antinuclear antibodies.

### 2.3. Cytometer

Immune complexes were analyzed on the flow cytometer, Cytomics FCM 500 from Beckman Coulter 22, Avenue des Nations CS 54359, 93420, Villepinte. It is equipped with 2 lasers 488 nm, 40 mW and 638 nm, 25 mW. It has the ability to analyze events on five colors: 525 nm, 575 nm, 620 nm, 675/695 nm, and 755 nm.

The sheath fluid for the cytometer was IsoFlow™ Sheath Fluid from Beckman Coulter, Part Number 8448010, an isotonic fluid at a pH 7.35-7.65, with Sodium Phosphate Dibasic, Sodium Fluoride, Diethylene Glycol Phenyl Ether, and 2-Phenoxyethanol.

A microparticle gate was established in agreement with the published method for characterization of circulating cell-derived microparticles by preliminary standardization experiments using a blend of size-calibrated 0.3, 0.5, 0.9, and 3 *μ*m fluorescent beads, Megamix®, Biocytex, Marseille, France [27, 28]. To enlarge the scale of fluorescent beads, in some experiments, Trucount beads (Beckton Dickinson) of 10 *μ*m were added.

A gate for DNA-anti-DNA immune complexes for each series of experiments was built around DNA-anti-DNA immune complexes observed in the aliquot of the pool of 15 SLE serum samples incubated with calf thymus DNA. See below for more details and for compensations.

### 2.4. Detection of Anti-dsDNA Antibodies by Flow Cytometry

Before taking a sample, the serum sample conserved at 4°C was mixed with a vortex shaker. 25 *μ*l of each serum sample was placed into two tubes. The first was incubated for one hour at 4°C with 200 *μ*l of calf thymus DNA diluted in PBS at 20 *μ*g/ml in order to detect the anti-dsDNA antibodies. In the other tube, 200 *μ*l of PBS was added in order to detect the circulating endogenous DNA-anti-DNA immune complexes. The DNA-anti-dsDNA antibody immune complexes were isolated by two different processes: (a) polyethylene glycol (PEG) at the final concentration of 3.5% precipitates and concentrates soluble immune complexes [29]. PEG had previously been used in the measurement of low-avidity anti-dsDNA antibodies, to precipitate the DNA-anti-dsDNA antibody immune complexes (IC-DNA) produced after incubation with DNA [30] and to isolate circulating IC-eDNA [13]. After adding the PEG to each tube, the mixture was incubated an additional 30 min. Finally, 1 ml of PBS was added for washing and centrifuged for 20 min at 4°C for 2860 g. The supernatant was sucked up and the pellet was kept. (b) To isolate cryo-insoluble ICs the process was similar to PEG precipitation except for the PEG. The cryo-insoluble IC are of the same nature as the cryoglobulins of the SLE serum samples [16]; the difference being that the cryoglobulins sediment within a few days; we accelerated this phenomenon by centrifugation. When serum samples are conserved at 4°C, before taking a sample for FCM, shaking is essential because IC-eDNA sediment.

On the pellet of the A or B process, 50 *μ*l of FITC-conjugated antiserum to human immunoglobulins was added and incubated for 30 minutes (the antiglobulin used was identical to the Crithidia luciliae indirect immunofluorescence test; see above for more details). After which, 400 *μ*l of ethidium bromide (EB) 10 *μ*g/ml was added and then analyzed by flow cytometry. For each tube, events were acquired at a fast speed, during 1 min.

When exposed to ultraviolet light, EB becomes fluorescent and emits an orange light of 605 nm wavelength. Its binding to double-stranded DNA increases the fluorescence intensity almost 20-fold. The fluorescence was detected on the sensors FL3 (620 nm) and FL4 (675). FL2 (575 nm) was not used because FITC and EB have fluorescence overlapping. FITC fluorescence (519 nm) was detected on the FL1 sensor (525 nm).

*A gate* to detect immune complexes (IC) was built around DNA-anti DNA IC observed in FCM for an aliquot of the pool of 15 SLE sera incubated with DNA. For each series of experiments, the parameters of this gate were defined by the side scatter, FITC fluorescence of the second antibody in FL1, and EB fluorescence in FL3 or FL4.

In FL1, *fluorescence compensations* were calculated with DNA labeled with EB; in FL3 and Fl4, compensations were calculated with FITC-labeled immune complexes without EB labeling.

### 2.5. Detection of Antibodies to Anti-dsDNA Antibodies and Soluble Nuclear and Cytoplasmic Antigens by Routine Methods

In the fluorescence enzyme immunoassay, IgG anti-dsDNA antibodies (EliA™-dsDNA) were detected on dsDNA plasmids derived from Escherichia coli coated to a solid support (dsDNA EliA™ well) and processed by a fully automated assay using UniCap100 (Pharmacia-Thermo Fisher Scientific). Results were expressed in international units (IU), and the threshold for a positive result was >15 IU/ml. Diagnostic sensitivity and specificity of EliA™-dsDNA had been compared to the Farr-RIA test and the CLIFT [31].

Anti-dsDNA antibodies by ultrasensitive Crithidia luciliae indirect immunofluorescence test (usCLIFT). Slides with Crithidia luciliae coated wells are provided by the company Theradiag (Marne-La-Vallée, France). Serum samples were tested at a single dilution of 1/10.

Smeenk et al. showed on 14 serum samples of SLE that the sensitivity of CLIFT increased from 29% positive samples with the standard test to 64% when using PBS 0.1 M NaCl instead of the classic PBS 0.14 M NaCl [30]. More recently, a highly sensitive detection of double-stranded DNA antibodies by a modified Crithidia luciliae immunofluorescence test was described [32, 33]. The authors claim to work with a “modified” buffer, without specifying the exact composition, because it is a trade secret (nDNA IFA plus; GA generic Assays GmbH). Following this work, we have previously developed an ultrasensitive test, the usCLIFT, a variant based on the use of a low ionic strength buffer, the “magic buffer” [34]. The serum samples were diluted 1/10 in the “magic buffer,” and then they were deposited on the slides coated with Crithidia luciliae, and after incubation, the slides were washed with this buffer. The “magic buffer” was prepared taking into account the work of Smeenk et al. [30]. One vial of PBS powder from Bio-Rad for immunofluorescence (pH 7.3, 11.34 g) was reconstituted in 1.5 l of distilled water instead of 1 l. The pH is not modified, but the ionic strength is lower than that of conventional PBS.

Antinuclear antibodies were detected by indirect immunofluorescence on HEp-2 cells EliA™ Symphony well, ANA screening for a mixture of human recombinant U1RNP (70 kDa, A, C), Ro (60 kDa, 52 kDa), La, Centromere B, Scl-70 and Jo-1 proteins, native purified Sm proteins, for the ImmunoCAP® 100, Thermo Fisher Scientific.

ELISA test for the determination of specific antibodies against nuclear and cytoplasmic antigens nRNP/Sm, Sm, SSA (Ro 60) Scl-70, Jo-1 ref EA 1590-1208-1G was purchased from Euroimmun/Bio Advance, Bussy Saint Martin 77600, France.

### 2.6. Statistics

Results of flow cytometry were analyzed in blind, the operator being uninformed of the patient's diagnosis and of the laboratory tests results. The *χ*^2^ and a two-tailed Fisher test were used for comparison of qualitative results and the Wilcoxon nonparametric test for quantitative results. The diagnostic sensitivity of a laboratory test was calculated as the ratio of the number of SLE patients with a positive test result to the total number of SLE patients tested. The diagnostic specificity was calculated as the ratio of the number control subjects with negative test results, to the total number of control subjects tested. For each test, the cutoff for positive results was determined by analysis of the area under the curve of the receiver operating characteristic (ROC) curve created by plotting the diagnostic sensitivity against 1 − specificity at various threshold settings. Qualitative agreement between two methods was calculated by the Cohen kappa statistic (*κ*).

## 3. Results

### 3.1. Development of the Detection of Anti-dsDNA Antibodies by Flow Cytometry (FCM)

**Figure 1:**
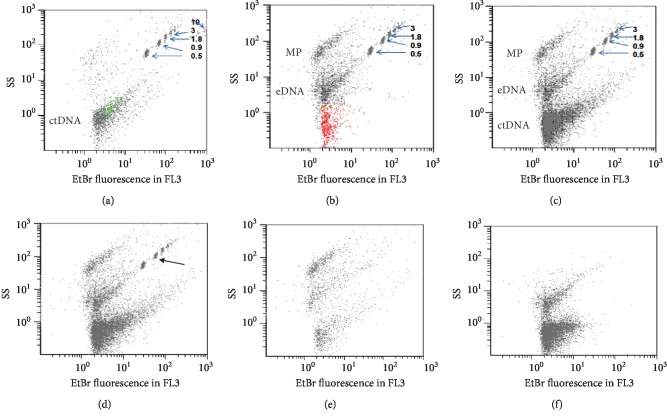
DNAse I and detergent sensitivity of serum particles detected by flow cytometry. The ordinate of histograms is the side scatter (SS) and the abscissa; the fluorescence of DNA stained by ethidium bromide (EB), measured at 620 nm (FL3). The discriminator was EB fluorescence; only events tagged by EB are shown. Upper histograms. (a) Calf thymus DNA (ctDNA) at 10 *μ*g/ml. (b) A SLE serum sample with anti-DNA antibodies containing endogenous DNA (eDNA) and microparticles (MPs). MPs may be stained with EB because they may contain DNA. (c, d) The same serum sample incubated with 20 and 40 *μ*g/ml ctDNA. (e) The serum sample after incubation with 20 *μ*g/ml ctDNA was treated with DNAse I or (f) treated with Tween 80 diluted 1/1000. The arrows indicate size-calibrated 0.5, 0.9, 3, and 10 *μ*m fluorescent beads. A microparticle gate was established using size-calibrated beads as described [27, 28].

Lyophilized calf thymus DNA (ctDNA) at 20 *μ*g/ml was used to detect anti-dsDNA antibodies. In FCM, ctDNA had very low light scatters. To improve its detection, DNA was stained by ethidium bromide (EB) (Figure 1(a)). The light scatters and EB staining of lyophilized ctDNA extended over a wide range of values because the size of ctDNA is heterogeneous. The fraction with the lower scatters overlapped the background noise. To reduce the number of events analyzed, that were not relevant to our study, we used a fluorescence threshold as “discriminator.” Only events tagged by EB were analyzed; events not tagged by EB were excluded. This reduces the background noise by 95%.

After incubation of serum samples with ctDNA, the resulting immune complexes ctDNA-antiDNA antibodies (IC-ctDNA) were identified by double labeling, EB staining for DNA, and for immunoglobulins FITC-conjugated antiserum to human immunoglobulins (IgG, IgA, IgM) (FITC-anti-Ig). IC-ctDNA had a light scatter and EB fluorescence is somewhat similar to ctDNA, but was easily distinguished because it was labelled by FITC-anti-Ig.

Because FITC-anti-Ig is neutralized by free immunoglobulins which are in excess, the immune complexes must be isolated, after which the antiglobulin can bind to the immune complexes. The cryo-insoluble IC-ctDNA or IC-ctDNA precipitated by polyethylene glycol (PEG) at 3.5% were isolated by centrifugation at 2860 g during 20 min at 4°C. Other methods to isolate ICs such as agarose resin, magnetic beads, or Staphylococcus aureus cells were unsuitable for this study.

In the absence of anti-dsDNA antibodies, 33% of the free ctDNA was present in the pellet because of a decrease in the solubility of lyophilized ctDNA under the physicochemical conditions that we used to promote the precipitation of ICs. When anti-dsDNA antibodies were present, the percentage of ctDNA in the pellet increased and reached 100% when the antibody titer was high.

### 3.2. Identification by FCM of PEG-Insoluble or Cryo-Insoluble Molecules

PEG precipitates in addition to ICs, different types of particles present in the serum samples (Figure 1(b)). The pellet of cryo-insoluble molecules was fragile, less abundant, and hardly visible to the eye. In return, the analysis of the histograms and the interpretation of the results were easier.

At least 3 kinds of serum particles were stained with EB in the centrifugation pellet of PEG-insoluble or cryo-insoluble molecules (Figures 1(b) and 1(c)). Particles smaller than 0.5 *μ*m in size as well as ctDNA were totally or partially destroyed by DNase I, but were resistant to detergents in moderate concentrations (Figures 1(e) and 1(f)). EB staining and sensitivity to DNase I confirmed that particles detected in some serum samples before incubation with ctDNA contained endogenous DNA (eDNA). Particles with a size greater than 0.5 *μ*m were resistant to DNase I, but destroyed with detergents suggesting that they had a lipid membrane and could be microparticles (Figures 1(e) and 1(f)). The IC-ctDNA and the IC-eDNA were smaller than 0.5 *μ*m; they did not overlap on the microparticles larger than 0.5 *μ*m (Figures 1(b)–1(d)).

When the reaction was performed at 37°C instead of 4°C, the amount of IC-ctDNA or IC-eDNA in PEG-insoluble and cryo-insoluble pellet was very significantly reduced.

### 3.3. Isolation and Identification of Different Types of IC-DNA

**Figure 2:**
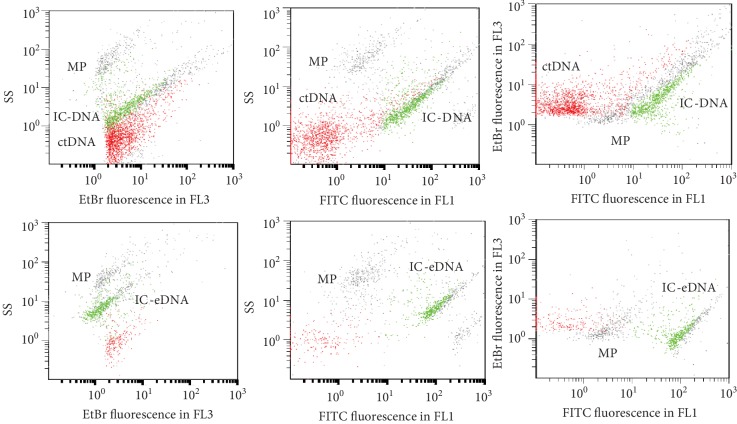
Detection of immune complexes DNA-anti-DNA antibodies (IC-DNA). In the upper histograms, a serum sample of SLE incubated with 20 *μ*g/ml ctDNA and in the lower histograms before incubation. SS is the side scatter; FL3 (620 nm) is fluorescence of DNA stained by EB and FL1 (525 nm) fluorescence due to antibody labeling by FITC-conjugated anti-human globulin. IC-eDNA, microparticles (MPs), and some free DNA already were present in the serum sample, but after incubation with ctDNA, the rate of IC-DNA increased significantly. The discriminator was EB fluorescence; only events stained by EB are shown. For each series of experiments, a gate was built around IC-DNA in order to quantify events. The parameters of this gate were defined by the SS, FITC fluorescence of the second antibody in FL1, and fluorescence of DNA stained by EB in FL3.

IC-ctDNA usually had a slightly lower light scatters than IC-eDNA but was insufficient to distinguish it. In addition, both types of IC overlapped for fluorescence with EB and with FITC-anti-Ig (Figure 2). Therefore, to obtain the exact amount of newly formed IC-ctDNA, the amount of IC-eDNA already present in the sample must be subtracted. For this reason, from each serum sample, two samples were taken; one was used to detect IC-eDNA. The other to detect anti-DNA antibodies was incubated with calf thymus DNA (ctDNA).

The scatters and EB staining of the immune complexes extend over a wide range of values; this could be explained on the one hand by the heterogeneity of DNA and on the other hand by the formation of DNA and antibody polymers of different sizes.

**Figure 3:**
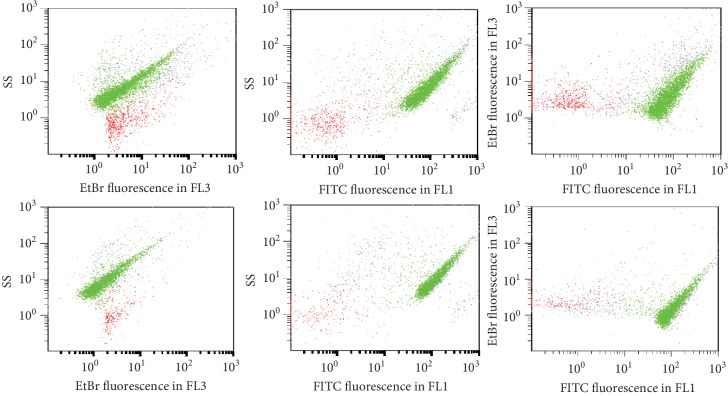
A serum sample of SLE with IC-eDNA and no detectable free anti-DNA antibodies. SS is the side scatter; FL3 is fluorescence of DNA stained by EB and FL1 fluorescence due to antibody labeling by FITC-conjugated antiglobulin. In the upper histograms, SLE serum sample incubated with ctDNA and in the lower histograms before incubation. IC-eDNA and some free DNA were already present in the serum, but after incubation with the ctDNA, the IC-DNA level did not increase significantly.

Out of the 63 serum samples of SLE patients, 21 (33%) had IC-eDNA. In some of these sera, free anti-dsDNA antibodies were hardly detectable or undetectable (Figure 3 and see below for more details).

**Figure 4:**
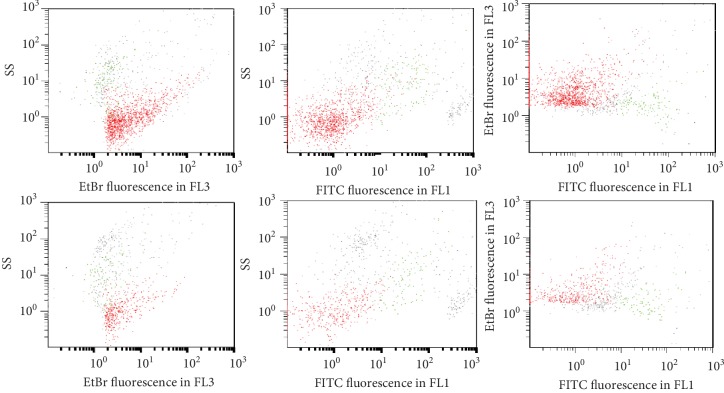
Control serum in the FCM test. In the upper histograms, control serum incubated with 20 *μ*g/ml ctDNA and in the lower histograms before incubation. Unlike SLE serum samples, no IC-DNA was detectable.

In the control serum samples, IC-eDNA were undetectable and the incubation with ctDNA did not generate IC-ctDNA (Figure 4).

### 3.4. Normalization and Quantitative Measurement of Anti-dsDNA Antibodies

**Figure 5:**
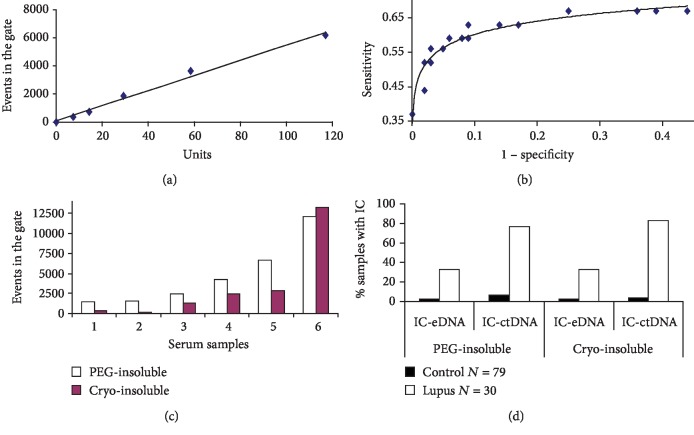
Quantitative measurement and normalization of anti-DNA antibodies and circulating immune complexes detected by flow cytometry (FCM). (a) A standard curve for quantitative measurements and normalization of anti-DNA antibodies. A pool of 15 SLE serum samples had been calibrated in international units (IU) against EliA™-dsDNA. In each series of tests, a standard 5-point curve was constructed by serial dilution of an aliquot of the pool. Each dilution was incubated with 20 *μ*g/ml of ctDNA in the same technical conditions than SLE serum samples. In the ordinate events in the gate of IC-DNA and in the abscissa anti-DNA units. (b) The cutoff for a positive IC result in lupus samples. The cutoff was determined by a receiver operating characteristic (ROC) curve created by plotting the diagnostic sensitivity against 1 − specificity at various threshold settings for lupus sera samples (*N* = 30) and control sera (*N* = 79) incubated with ctDNA. The curve shown was observed with the PEG-insoluble immune complexes; a similar curve was observed with the cryo-insoluble immune complexes. (c, d) Comparison of PEG-insoluble to cryo-insoluble immune complexes (IC). (c) Number of events observed in the gates for IC-DNA, by running the cytometer at a fast rate for 1 minute. Serum samples were incubated with 20 *μ*g/ml ctDNA. 1-3 control serum samples, 4-6 lupus serum samples. The events observed with the control serum samples were due to the background of the FCM test. (d) Percentage of samples with IC above the cutoff in 30 SLE serum samples with anti-DNA antibodies detected by the routine methods and in 79 control serum samples. Two types of IC are shown: IC with endogenous DNA (eDNA) spontaneously present in SLE serum samples and IC generated after incubation of samples with ctDNA.

For quantitative measurement of anti-dsDNA antibodies and of IC-DNA by the FCM test, a pool of 15 serum samples of SLE patients had been calibrated in international units (IU) against EliA™-dsDNA, a fluorescent enzyme immunoassay to detect anti-dsDNA antibodies. The pool was aliquoted and stored at -40°C. In each series of tests, a standard 5-point curve was constructed by serial dilution of an aliquot of the pool (1/1, 1/2, 1/4, 1/8, 1/16). Each dilution and a control tube with buffer were incubated with ctDNA in the same technical conditions than SLE sera (Figure 5(a)). The number of events in the gate decreased in proportion to the dilution. In contrast, the mean fluorescence intensity (MIF) of events in the gate remained stable for the first 3 dilutions and then decreased. The number of events in the gate correlated better with dilution than the MIF.

The cutoff for a positive IC result in lupus samples was determined by a receiver operating characteristic (ROC) curve created by plotting the diagnostic sensitivity against 1 − specificity at various threshold settings for lupus sera samples (*N* = 30) and control sera (*N* = 79) incubated with ctDNA. One ROC curve was constructed with PEG-insoluble IC-DNA and another one with cryo-insoluble IC-DNA (Figure 5(b)). Thereafter, diagnostic sensitivity and specificity for the detection of anti-dsDNA antibodies and circulating IC-eDNA were compared; the two processes gave similar results (Figures 5(c) and 5(d)). Therefore, both processes were used.

**Figure 6:**
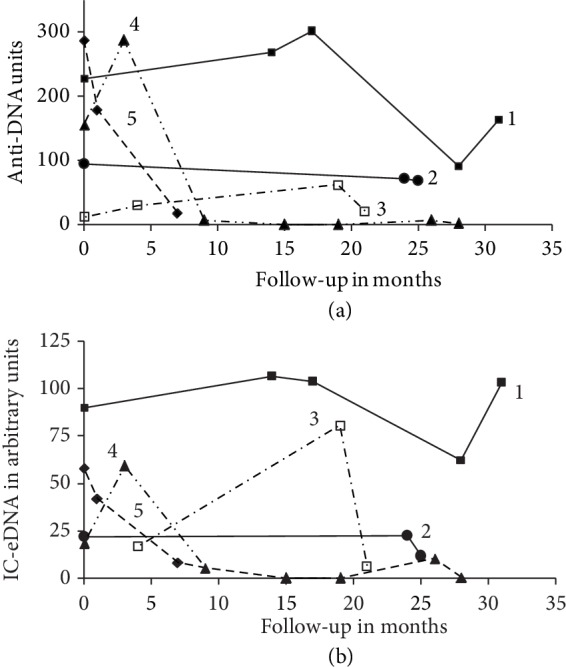
Variation of the anti-DNA antibody levels in the top figure and circulating DNA immune complexes (IC-eDNA) in the bottom figure detected by FCM during patient follow-up. Five representative patients (1-5) are shown. All patients had a medical treatment. In FCM, one arbitrary unit for IC-eDNA was defined as equivalent to the number of events observed with one unit of anti-DNA antibodies incubated with calf thymus DNA, in the gate built around DNA immune complexes.

Standardization made it possible to follow-up and compare the results observed on serum samples taken at different dates from the same patient. The levels of anti-DNA antibodies and circulating IC-eDNA varied during follow-up; all these patients had a medical treatment which had the effect of lowering the levels of antibodies (Figure 6).

### 3.5. Comparison of the FCM Test to Routine Anti-dsDNA Antibody Detection Tests in a Cross-Sectional Analysis

In 63 SLE patients and 87contol patients, the diagnostic sensitivity of the FCM test was lower than that the ultrasensitive Crithidia luciliae indirect immunofluorescence test (usCLIFT) and the EliA™-dsDNA test. However, the latter with a threshold of 15 IU (recommended by the manufacturer) was not very specific (Sp = 0.75). With a threshold of 30 IU (according to the SLICC criteria which recommends for the diagnosis of SLE, to take twice the threshold value of positivity for the ELISA and from the findings of the comparative studies on dsDNA antibody testing with the EliA™-dsDNA test) [2, 35], the sensitivities of the EliA™-dsDNA test and the FCM test were comparable, and their specificity was as high as the usCLIFT (Table 1).

In the 21 SLE serum samples containing IC-eDNA, the anti-dsDNA antibodies were hardly detectable or undetectable: 13 (62%) serum samples, free anti-dsDNA antibodies were undetectable by the FCM test (Figure 3); among them, 8 (38%) were negative or borderline (between 13 and 26 IU) in the EliA™-dsDNA and 4 (19%) were negative in the usCLIFT.

In 29 patients with incomplete SLE (<4 ACR criteria) or lupus nephritis and 3 drug-induced lupus (DIL), anti-DNA antibodies and IC-eDNA were detected in the serum samples by the FCM test. In these patients, anti-DNA antibodies were also detected by the routine tests (Table 2).

## 4. Discussion

Detection of anti-dsDNA antibodies by FCM had a diagnostic sensitivity and a specificity comparable to the routine test EliA™-dsDNA test with a threshold of positivity at 30 IU/ml. However, the sensitivity of the usCLIFT was higher. The usCLIFT we used was different from the classical CLIFT test because the buffer had a weaker ionic strength and this leads to a strong increase in sensitivity without decrease in specificity [30, 32–34].

The percentage of SLE patients with circulating anti-dsDNA antibodies is underestimated because all patients in this study were under treatment. The medication induces antibodies diminishing or even antibodies disappearing (Figure 6). Eight SLE patients with undetectable dsDNA antibodies in the blood samples during this study had been regularly monitored; they already had antibodies that disappeared within 2-50 months (median 19 months) after the start of treatment.

In addition to detect anti-DNA antibodies, FCM offers the ability to detect circulating IC-eDNA which is not possible with routine tests. No correlation was observed between circulating IC-eDNA and the level of anti-dsDNA antibodies detected by usCLIFT or EliA™-dsDNA test. Our results are in accord with those published [13, 36]. Patient 3 in Figure 6 initially had the lowest level of anti-DNA antibodies, but achieved a high level of IC-eDNA in the 20th month of follow-up. In the majority of patients, an inverse relationship was found between the levels of circulating IC-eDNA and anti-dsDNA antibodies [15]. Thus, in samples initially having high levels of anti-dsDNA antibodies, after formation of ICs with eDNA, very little free anti-dsDNA antibodies can remain [15]. In the extreme, these serum samples can give false negative results in the detection of anti-dsDNA antibodies by the routine tests as well as the FCM test (Figure 3), because the results of the tests depends on the amount of free antibody present in the serum sample. The detection of IC-eDNA by flow cytometry offers an interesting alternative that can be used routinely to avoid this trap.

The dual measurement of free antibodies and bound antibodies in the IC-eDNA may allow more accurate diagnosis and monitoring of relapses in patients with SLE. We have not been able to study the link between the variation of these serological parameters and the disease activity during follow-up. Although we had at least 2 samples per patient, we had not planned to collect clinical data at the time of each sample; we had the clinical diagnosis when the study was completed. In addition, the patients included in the study were treated. A prospective study would be needed to determine this link. On the other hand, we show that incomplete lupus patients had anti-DNA antibodies and IC-eDNA-like SLE patients (Table 2), which sustains the inclusion of some of these patients in SLE according to SLICC criteria [2].

Although the FCM test is promising, it has disadvantages, and it is more expensive and time-consuming than routine assays; moreover, the interpretation of the results is more complex. This complexity comes from the great diversity of particles present in serum or plasma. To reduce the diversity of particles analyzed and select those that are concerned by this study, we used as a “discriminator” a fluorescence threshold; only events marked by EB were analyzed, and events not marked by EB were excluded.

Three types of serum particles were PEG-insoluble or cryo-insoluble and stained with EB. Two out of three were totally or partially destroyed by DNase I. Partial resistance might be explained by the fact that when DNA is included in a more complex structure like such as the nucleosome or chromatin or IC-eDNA, this confers DNase resistance [37]. The third type particle was resistant to DNase I and had microparticle (MP) characteristics, a size greater than >0.5 *μ*m, and was detergent sensitive. A simple method to discriminate between IC- and MP-related events by FCM consists in using low concentrations of detergent (Triton X-100, Tween 20) which lyses the MP, while ICs or other protein complexes are insensitive [20].

IC-DNA could be isolated because they were PEG-insoluble and cryo-insoluble. Soluble ICs consist of 2 to 10 macromolecules, whereas insoluble ICs form colloidal precipitates, as is the case of cryoglobulins in the serum of patients [38]. The cryo-insoluble IC-eDNA are of the same nature as the cryoglobulins of the SLE sera, the difference being that the cryoglobulins sediment within a few days [16], and we accelerated this phenomenon by centrifugation. When serum samples are conserved at 4°C, before taking a sample for FCM, shaking is essential because IC-eDNA sediment.

Soluble ICs lose their solubility for a given antigen/antibody ratio, temperature, ionic strength, pH, or indirect immune labelling by secondary antibodies (anti-human globulin) [37]. In our study, when the reaction was performed at 37°C instead of 4°C, the amount of IC-DNA recovered was very significantly reduced for both processes.

PEG had previously been used in the measurement of low-avidity anti-dsDNA antibodies, to precipitate the DNA-anti-dsDNA antibody ICs produced after incubation of SLE sera with DNA [30] and to isolate circulating IC-eDNA [13]. The volume of the PEG precipitate was greater than the centrifugation pellet of cryo-insoluble molecules, but the background in FCM was greater because PEG precipitates in addition to IC, different types of particles present in the serum.

EB is a nonspecific intercalating molecule; it binds to both double-stranded (ds) DNA and dsRNA as well as to double-stranded regions in single-stranded nucleic acids in order to form fluorescent complexes. Since EB can label RNA, it may be questionable that some of the ICs we have detected contain RNA instead of DNA. The particles stained by EB were destroyed by DNAse I, confirming that these particles contained DNA. Moreover, circulating RNases destroy circulating RNA with some exceptions. Circulating small noncoding RNAs (snc RNAs) such as microRNAs (miRNAs), piwi-interacting RNAs (piRNAs), small nucleolar RNAs (snoRNAs), tRNAs, U1, and Y1 RNAs were found to be stable in biofluids. They are transported by membrane-derived vesicles (exosomes and microparticles), lipoproteins, and ribonucleoprotein complexes which protect them from circulating RNases [39]. In patients with SLE, antibodies against ribonucleoproteins containing U1RNA (Sm, RNP, and U1RNP68 autoantigens) or Y1RNA (SSA/Ro52, SSA/Ro60, and SSB/La autoantigens) and the ICs containing these ribonucleoproteins circulate [40]. These snc RNAs are present in serum in too small amounts, they are single-stranded with some exceptions (U1 and Y1 in some portions they fold to be double-stranded), and their size is too small (<200 bases for U1 and Y1, <100 bases for other sncRNAs) to form fluorescent complexes detectable by FCM. Knowing that the lyophilized ctDNA is double-stranded and contains at least 150 times more nucleotides (average molecular weight M(w) = 8418000, equivalent to 27600 bases) [41], and that with EB, the signal in our technical conditions is just above the background noise. To label DNA, more specific fluorescent stains as 4′,6-diamidine-2-phenylindole (DAPI), Hoechst dyes, and SYBR green I are available. Each dye has advantages and drawbacks that depend on the context of use [42]. Any small molecule capable of binding DNA with high affinity is a possible mutagen. That is why we handled EB with great care (for more details, see Patients and Methods, section 2.2) [26, 43]. Work should be undertaken to substitute for EB another less mutagenic dye that should not compete with the binding of anti-DNA antibodies and whose fluorescence would not be quenched by the presence of antibodies on DNA.

Free DNA could be detected in serum samples, but quantification was not accurate because FCM can detect DNA when it has a high molecular weight comparable to lyophilized ctDNA. The detection of the latter is limited by the performance of the cytometer; indeed, light scatters and EB staining are just above the background noise. A second hurdle was that we had worked on serum samples which is not advisable to detect free DNA. Differences in DNA concentration between serum samples and plasma samples were described [44]. DNA is released in serum samples during clotting. In normal plasma samples, no DNA was detected in contrast to serum samples. It was suggested that the study of IC-eDNA as well as the detection of anti-nDNA antibodies by the sensitive Farr technique could be performed more reliably on plasma rather than serum [44]. However, since this publication, these suggestions have not been taken into account for the routine anti-dsDNA antibody immunoassays; serum samples are still used for the detection of anti-dsDNA antibodies. This is perhaps related to the possible fibrinogen interference present in plasma samples and the risk of formation of microclots in immunoassays.

In conclusion, we find that flow cytometry detects anti-dsDNA antibodies with a diagnostic sensitivity and specificity almost comparable to routine tests such as the usCLIFT and the EliA™-dsDNA. In addition, it detects the circulating immune complexes of endogenous DNA-anti-dsDNA antibodies (IC-eDNA). In some serum samples, all the DNA antibodies can be neutralized by the endogenous DNA; they are no longer detectable by routine tests, which might suggest the absence of anti-dsDNA antibodies. In such cases, the detection of circulating IC-eDNA could help to serologic diagnose and track relapses in patients with SLE; flow cytometry is able to do this detection.

## Figures and Tables

**Table 1:** Clinical sensitivity and specificity of anti-dsDNA detection tests in a cross-sectional analyses. 63 serum samples of SLE are classified according to the results observed on the usCLIFT.

	Nb of positive sera (%)				
	usCLIFT positive	EliA™-dsDNA		FCM test	
		Cutoff > 15 IU^∗^	Cutoff > 30 IU	IC-ctDNA^+^	IC-eDNA^
Control					
*N* = 87	1 (1.1%)	22 (25%)	4 (4.6%)	5 (5.7%)	2 (2.3%)
SLE^&^					
*N* = 63	46 (73%)	48 (76%)	30 (48%)	35 (56%)	21 (33%)
usCLIFT-					
*N* = 17		8 (47%)	1 (5.9%)	3 (18%)	4 (24%)
usCLIFT+					
*N* = 46		40 (87%)	29 (63%)	32 (70%)	17 (37%)
Sensitivity^§^	0.73	0.76	0.48	0.56	0.33
Specificity	0.99	0.75	0.95	0.94	0.98

^∗^The threshold of positivity according to the manufacturer is 15 IU/ml; however, according to the SLICC criteria and published results, the threshold in ELISA for the diagnosis of SLE should be twice the threshold of positivity [2, 35].^+^IC-ctDNA, *p* < 0.001, for the concordance with usCLIFT or EliA™-dsDNA > 30 IU and *p* = 0.044 with EliA™-dsDNA > 15 IU. ^No statistically significant relationship between the presence of circulating IC-eDNA and the results of anti-DNA detection by usCLIFT or EliA™-dsDNA. ^&^The results of all tests including IC-eDNA are statistically significant between SLE sera and control samples, *p* < 0.001. ^§^In order not to introduce any difference in the sensitivity and the specificity due to the labeling of the different antibody isotypes, the same FITC-conjugated antiserum to human immunoglobulins (IgG, IgA, IgM) was used in the FCM test and in the usCLIFT. The sensitivity of the usCLIFT was higher than the FCM test and EliA™-dsDNA > 30 IU, *p* < 0.01.

**Table 2:** Anti-DNA antibodies in patients with lupus nephritis or incomplete lupus erythematosus^∗^ or drug-induced lupus.

Patients	Nb of positive sera (%)				
	usCLIFT positive	EliA™-dsDNA		FCM test	
		Cutoff > 15 IU	Cutoff > 30 IU	IC-ctDNA	IC-eDNA
Lupus nephritis					
*N* = 9	4 (44%)	6 (67%)	3 (33%)	2 (22%)	4 (44%)
Chronic CL^					
*N* = 14	3 (21%)	6 (43%)	2 (14%)	5 (36%)	3 (21%)
Subacute CL^&^					
*N* = 6	2 (33%)	3 (50%)	2 (33%)	3 (50%)	2 (33%)
DIL^§^					
*N* = 3	1 (33%)	3 (100%)	0 (0%)	2 (66%)	1 (33%)

^∗^Patients with <4 ACR criteria, but those with anti-DNA antibodies satisfied SLICC criteria for SLE [2, 22, 23]. ^Chronic cutaneous lupus. ^&^Five patients out of 6 with subacute cutaneous lupus had anti-Ro60 antibodies. ^§^Drug-induced lupus; 2 patients were treated by infliximab, and another one by anticancer chemotherapy.

## Data Availability

The data used to support the findings of this study are included within the article.

## Abbreviations

eDNA: Circulating endogenous DNA
ctDNA: Calf thymus DNA
IC: Immune complex
IC-eDNA: Immune complex of eDNA-anti-DNA antibodies
EB: Ethidium bromide
FCM: Flow cytometry
PEG: Polyethylene glycol
EliA™-dsDNA: ELISA to detect anti-DNA antibodies
usCLIFT: Ultrasensitive Crithidia luciliae indirect immunofluorescence test.
